# Treosulfan is a safe and effective alternative to busulfan for conditioning in adult allogeneic HSCT patients: Data from a single center

**DOI:** 10.1002/cam4.7292

**Published:** 2024-05-16

**Authors:** Ant Uzay, Yasemin Gündoğdu, Barış Koşan, Tuğba Yetiş, S. Sami Kartı

**Affiliations:** ^1^ School of Medicine, Department of Hematology and Stem Cell Transplantation Acıbadem Mehmet Ali Aydınlar University Istanbul Turkey; ^2^ School of Medicine, Department of Internal Medicine Acıbadem Mehmet Ali Aydınlar University Istanbul Turkey; ^3^ School of Medicine, Clinical Nursing Acıbadem Mehmet Ali Aydınlar University Istanbul Turkey

**Keywords:** busulfan, graft versus host disease, stem cell transplantation, toxicity, treosulfan

## Abstract

**Introduction:**

Type of conditioning regimen impacts the outcome of patients who undergo allogeneic HSCT since graft versus host disease (GVHD), infections, regimen related toxicities (RRT) are important causes of post‐transplant mortality. Despite the RRT profile of busulfan, it is frequently used worldwide. Treosulfan has advantages in terms of dose of administration, lower incidence of sinusoidal obstruction syndrome and lower neurotoxicity. We retrospectively investigated outcomes of patients who underwent allogeneic HSCT with treosulfan or busulfan based conditioning regimens in our institution.

**Methods:**

Treosulfan was administered to 94 patients while 85 patients received busulfan. Our outcomes were RRT, chronic and acute GVHD, relapse related mortality (RRM), non‐relapse mortality, and fungal infection. The clinical follow up data, regarding the primary and secondary endpoints of our study, of the patients who received treosulfan or busulfan based conditioning regimens were statistically analyzed.

**Results:**

The median follow‐up was 14 months for the treosulfan group while it was 11 months for the busulfan group (*p* = 0.16). RRT was 11.7% and 7.1% for treosulfan and busulfan respectively. The incidence of extensive chronic GVHD was less frequent in the treosulfan group compared to the busulfan group (15.7% vs. 32.1%) (*p* < 0.001). The incidence of acute GVHD (Grade 3 or higher) was 32.2% in the treosulfan group while it was 31.6% in the busulfan group. The RRM was 17% in the treosulfan group while it was 34% in the busulfan group. The non‐relapse mortality was 35.5% and 29.4% in the treosulfan group and in the busulfan group respectively (*p* = 0.962).

**Conclusion:**

Treosulfan, with a lower RRM, lower chronic GVHD incidence and with a similar RRT profile appears to be a safe alternative to busulfan.

## Abstract 2

Herein, we describe the outcomes and compare results of patients who underwent matched and haploidentical allogeneic hematopoietic stem cell transplantation (HSCT) with treosulfan based or busulfan based reduced intensity, non‐myeloablative and myeloablative conditioning regimens in our institution. As busulfan has certain side effects and requires meticulous follow‐up during HSCT, we wished to convey our experience with a similar patient group who were administered treosulfan. Our aim was to describe challenges and outcomes which occurred while using both drugs for conditioning and finally to deliver the message that treosulfan is as safe and effective as busulfan.

## 1 INTRODUCTION

Allogeneic stem cell transplantation is a potentially curative treatment option in many hematological malignancies and inborn errors which otherwise have extremely poor prognosis. The main goal of the allogeneic transplantation in the setting of the hematological malignancy is the eradication of remnant malignant cells by an effective conditioning regimen that promotes the establishment of a healthy immune reconstitution which also has a prominent graft versus tumor effect. The achievement of that goal is frequently compromised by major complications that are specific to the allogeneic transplantation itself.

Many transplant patients suffer from conditions like life threatening infections, acute and chronic graft versus host disease (GVHD), organ and tissue toxicities due to the side effects of the preparative regimens and early disease relapse, which constitute the majority of the causes of mortality after allogeneic transplantation.^1^

Recent developments in donor selection, the advent of reduced toxicity and non myeloablative conditioning (MAC) regimens have led to reduced rates of complications.^2^ This in turn has created an opportunity for many frailed and advanced age patients who otherwise would not be fit enough for an allogeneic transplant. Despite the vast experience in the past two decades, none of the currently approved conditioning regimens that are used provides perfect solutions to the complications seen after allogeneic transplantation and there is still much room for improvement.

Treosulfan is an alkylating agent which was initially approved for the treatment of ovarian cancer and is currently used in combination with other drugs for the conditioning regimens predominantly in pediatric patients.^3^ The use of treosulfan in the adult transplant population was initially limited to patients who underwent reduced intensity conditioning (RIC).^4^ Given the excellent myelosuppressive and immunosuppressive effects with low proinflammatory cytokine release properties, the interest toward this drug has increased dramatically in the past decade.^5^ Despite the increased use of treosulfan based conditioning regimens, especially in Europe, data is limited regarding its efficacy compared to standard myeloablative regimens and experience with this drug in unrelated and haploidentical transplants is insufficient.

Conditioning regimens with busulfan have been linked to complications like convulsions, sinusoidal obstruction syndrome (SOS), toxicity and GVHD.^5^ Despite the reported advantages of treosulfan, busulfan is still commonly used as a backbone of allogeneic HSCT conditioning regimens. Lower incidence of regimen related toxicity, transplant related toxicity and higher overall survival has been reported in some studies yet extensive data is needed.^3^ Herein this study we share and compare the results of our single center experience with treosulfan and busulfan based conditioning regimens used in adult patients with hematological malignancies who underwent matched related or unrelated haploidentical stem cell transplantation.

## 2 METHOD

### 2.1 Study design and data collection

This is a retrospective, single center analysis presenting real life data of adult patients diagnosed with hematological malignancies, who underwent allogeneic transplantation between September 2016 and November 2022 with conditioning regimens that included treosulfan or busulfan. Data were collected from the institution's electronic records, which offers access to all laboratory, radiology, genetic and pathology results as well as every physicians or nurse's follow‐up. This study was approved by the Institutional Review Board of Acıbadem Mehmet Ali Aydınlar University, School of Medicine (ATADEK) with the decree number 2023‐03/84 and was conducted in accordance with the Declaration of Helsinki and Good Clinical Practice guidelines. All patients provided written informed consent authorizing the use of their personal information for research purposes.

Eligibility criteria included all patients diagnosed with hematological malignancies which were: acute myeloid leukemia (AML), myelodysplastic syndrome (MDS), acute lymphoblastic leukemia (ALL), Non‐Hodgkin's lymphoma (NHL), Hodgkin's disease (HD), chronic myeloproliferative disorder (CMPD), chronic myeloid leukemia (CML), blastic plasmacytoid dendritic cell neoplasm (BPDCN), chronic lymphocytic leukemia (CLL) who underwent MAC or reduced intensity conditioning (RIC) allogeneic transplantation from matched related, matched unrelated or haploidentical donor.

All of the grafts were peripheral blood stem cells and were T cell replete. Ex vivo T cell depletion was not allowed. In vivo T cell depletion was done using anti‐human T‐lymphocyte immunoglobulin (ATG) (Grafalon) in patients with CMPD and some of the patients undergoing matched unrelated transplantation. HLA typing was based on high‐ resolution typing of class I and class II HLA antigens. Variables collected included recipient and donor characteristics which define pre‐transplant risk factors, disease features, transplant related factors such as anti‐infective and GVHD prophylaxis, total doses used in the conditioning regimen, and outcome variables. All the patients in the busulfan group had high SOS risk according to the European Group for Blood and Marrow Transplantation (EBMT) Handbook and were given concurrent defibrotide.^6^

Busulfan based conditioning regimens have been standard practice in our country however, after treosulfan became more accessible in 2019, we started to use treosulfan based regimens more frequently in our center. In accordance with regulations, we applied to the Ministry of Health for the acquisition of treosulfan with their approval for each patient. After 2019 we used busulfan only when our application was declined. Patients receiving busulfan who were considered high risk according to the EMBT handbook were given defibrotide prophylaxis intravenously with a dose of 6.25 mg/kg every 6 h, as suggested by the FDA.^6^, ^7^ In our unit, concurrent clonazepam was administered with busulfan for seizure prevention as neurotoxicity prophylaxis is recommended by various studies.^8^ Anti‐epileptics were not administered with treosulfan as it is considered a safer drug in terms of the neurotoxicity profile.

### 2.2 Conditioning regimens

MAC regimens were defined as those which included cyclophosphamide 120 mg/m^2^ of total dose.^9^ There are no internationally recognized strict criteria for treosulfan based MAC, and our center uses a combination regimen with a total dose of 120 mg/m^2^ fludarabine and a total dose of 42 mg/m^2^ treosulfan as a myeloablative regimen based on the previous studies.^10^ Melphalan and total body irradiation doses used to define MAC are not mentioned here as none of the patients received the mentioned treatments. The remaining conditioning regimens where treosulfan was used in lower doses and was combined with thiotepa or fludarabine were defined as reduced intensity conditioning (RIC). The total treosulfan dose was 30 mg/m^2^ combined with a total dose of 120 mg/m^2^ fludarabine for patients who were given non‐myeloablative (NMA) conditioning (Table 1). The total myeloablative dose of busulfan was 12.8 mg/kg (3.2 mg/kg/day) while the total dose used in non myeloablative regimen was 6.4 mg/kg combined with fludarabine. ATG was used at doses of 10 mg/kg/day for three consecutive days prior to matched unrelated donor (MUD) transplantation. GVHD prophylaxis varied according to the transplant type but mostly it consisted of cyclosporine (CsA) and methotrexate (MTX) or tacrolimus + mycophenolate mofetil (MMF)/post‐transplant cyclophosphamide (PTCy) in haploidentical transplant.

**TABLE 1:** Drug doses administered for the conditioning regimens.

	Treosulfan	Busulfan
MAC*	Treosulfan (14 mg/m^2^/day‐3 days)/Fludarabine (30 mg/m^2^/day‐5 days) Treosulfan (14 mg/m^2^/day‐3 days)/Thiotepa (5 mg/kg/day‐2 days)	Busulfan (3.2 mg/kg/day‐4 days)/Cyclophosphamide (60 mg/kg/day‐2 days) Busulfan (3.2 mg/kg/day‐4 days)/Fludarabine (30 mg/m^2^/day‐5 days)
NMA	Treosulfan (10 mg/m^2^/day‐3 days)/Fludarabine (30 mg/m^2^/day‐5 days) Treosulfan (10 mg/m^2^/day‐3 days)/Thiotepa (5 mg/m^2^/day‐2 days)	Busulfan (3.2 mg/kg/day‐2 days)/Fludarabine (30 mg/m^2^/day‐5 days)

Abbreviations: MAC, myeloablative conditioning; NMA, nonmyeloablative conditioning.

* All of the MAC regimens of Treosulfan were regarded as RIC regimens.

### 2.3 Exclusion criteria

Patients who received TBI, patients with low SOS risk who did not receive concurrent defibrotide, and patients with second transplantation were excluded from the study.

### 2.4 Study endpoints

The primary endpoints of this study were regimen related toxicity (RRT) and non‐relapse mortality (NRM) within 6 months. Secondary endpoints were relapse related mortality (RRM), total days of hospitalization, day of thrombocyte (>20.000 without transfusion) and neutrophil (>500 without G‐CSF) engraftment, incidence of engraftment failure, incidence of SOS, occurrence of severe systemic infection, incidence of opportunistic infections such as of fungal infection, CMV reactivation, and also the incidence of acute GVHD higher than Grade II and extensive chronic GVHD.

### 2.5 Evaluation of outcomes

Disease relapse was defined according to standard hematologic criteria. Non‐relapse mortality (NRM) was defined as death from any cause in the absence of prior disease recurrence within 6 months after transplantation. The cause of NRM was subcategorized as sepsis, GVHD, engraftment failure, non‐infectious CNS complication and multi‐organ failure. As for the RRT, only events of Grade 2 or higher were taken into account in order to give emphasis on the serious side effects. Conditioning regimen related toxicity was assessed using the Seattle criteria.^11^ The types of RRT was categorized as cardiac (arrhythmia, heart failure), CNS (intracranial hemorrhage, epilepsy, severe neuropathy), renal (acute or chronic renal failure), hepatic (hepatotoxicity), pulmonary (non GVHD related progressive decline of forced expiratory volume in the first second), hemorrhagic cystitis and combined (any of the two listed). The time frame of the toxicities attributed to the preparative regimen was from the start of the conditioning until 30th day after transplantation. This time frame was extended to the 100th day post‐transplant for pulmonary complications. The rationale to chose the 30th day for non‐pulmonary complications and the 100th day for pulmonary complications was due to the data extracted from the meta analysis of Danylsenko et al. and study of Nagler et al.^12^, ^13^ The cause of death of the patients who experienced relapsed disease at any time before death was considered relapse related. Risk stratification and grading of acute and chronic GVHD was done according to Minnesota criteria and the NIH grading system respectively.^14^, ^15^, ^16^ The revised Baltimore criteria was used for the diagnosis of SOS.^16^ SIRS, sepsis or infections that led to an ICU transfer (Common Terminology Criteria for Adverse Events (CTCAE) Grade 3 or higher grades) were considered for the criteria of systemic infection.^17^ Guidelines of The European Organization for Research and Treatment of Cancer were used for the diagnosis of invasive fungal disease.^18^ Complete donor chimerism was defined when chimerism of >98% was reached and chimerism values were calculated via short tandem repeat polymerase chain reaction and fluorescent in situ hybridization for sex matched and sex‐mismatched donor respectively.

### 2.6 Statistical analysis

The conformity of the continuous variables to the normal distribution according to the groups were evaluated with the Shapiro–Wilk normality test. Normally distributed continuous variables were compared between groups using independent samples Student *t*‐test with Levene's test of homogeneity. Mann‐Whitney *U*‐test was performed to compare non‐normally distributed continuous variables between groups. Pearson, Yates' continuity correction, Fisher's exact test and Monte‐Carlo Exact chi‐square analyzes were used to compare categorical variables between groups. Survival curves were calculated using the Kaplan–Meier method, and the differences were analyzed by the log‐rank test. A value of *p* < 0.05 was considered to indicate statistical significance. All analyzes were performed using the IBM SPSS Statistics Version 25 package program.

## 3 RESULTS

### 3.1 Patient characteristics

Characteristic features and demographic data is summarized in Table 2. This study included 179 patients with hematological malignancies of which 94 were given treosulfan and 85 were given busulfan for the conditioning regimens. Homogenous distribution among two groups with regards to gender, age, type of transplant, type of regimen and comorbidity types was observed. While the distribution among patients with AML and ALL was homogeneous between two groups, patients with MDS, HL, NHL and CML were more frequent in the treosulfan group.

**TABLE 2:** Baseline patient characteristics.

	Treosulfan *n* = 94	Busulfan *n* = 85	*p*‐value
Male *n*, (%)	49 (52.1)	50 (58.8)	0.36
Female *n*, (%)	45 (47.9)	35 (41.2)	
Age, median	41	44	0.42
Malignancy type			
AML *n*, (%)	41 (43.6)	46 (54.1)	
MDS *n*, (%)	10 (10.6)	3 (3.5)	<0.001
ALL *n*, (%)	21 (22.3)	21 (24.7)	
NHL *n*, (%)	12 (12.8)	5 (5.9)	
HD *n*, (%)	7 (7.4)	0 (0)	<0.001
CMPD *n*, (%)	0 (0)	8 (9.4)	<0.001
CML *n*, (%)	3 (3.2)	0 (0)	<0.001
BPDCN *n*, (%)	0 (0)	1 (1.2)	
CLL *n*, (%)	0 (0)	1 (1.2)	
Transplant type			
Full match sibling *n*, (%)	52 (55.3)	54 (63.5)	0.53
MUD *n*, (%)	19 (20.2)	14 (16.5)	
HAPLO *n*, (%)	23 (24.4)	17 (20)	
Conditioning Intensity			
Myeloablative *n*, (%)	79 (84)	66 (77.6)	36
Nonmyeloablative /RIC* *n*, (%)	15 (16)	19 (22.4)	
Remission status before transplant			
Complete remission [CR1] *n*, (%)	56 (59.5)	29 (34.1)	6
CR2 *n*, (%)	21 (22.3)	38 (44.7)	
> CR2 *n*, (%)	4 (4.2)	4 (4.7)	
Partial remission *n*, (%)	7 (7.4)	10 (11.7)	
Active disease *n*, (%)	6 (6.3)	4 (4.7)	
GVHD prophylaxis			
CsA/MTX *n* (%)	34 (36.1)	47 (55.2)	24
CsA/MMF/PTCy *n* (%)	25 (26.5)	18 (21.1)	
TACRO/MTX *n* (%)	6 (6.3)	3 (3.5)	
TACRO/MMF/PTCy *n* (%)	12 (12.7)	2 (2.4)	
CsA/MTX/ATG *n* (%)	17 (18)	15 (17.6)	

Abbreviations: ALL, acute lymphoblastic leukemia; AML, acute myeloid leukemia; ATG, anti‐thymocyte globulin; BPDCN, blastic plasmacytoid dendritic cell neoplasm; CLL, chronic lymphocytic leukemia; CML, chronic myeloid leukemia; CMPD, chronic myeloproliferative disorder; CsA, cyclosporine; GVHD, graft‐versus‐host‐disease; HAPLO, haploidentical; HD, Hodgkin Lymphoma; MDS, myelodysplastic syndrome; MTX, methotrexate; MUD, matched unrelated donor; NHL, non‐hodgkin lymphoma; PTCy, post‐transplant cyclophosphamide.

* RIC is applicable for treosulfan only.

The majority (59.2%) of the transplantations were from a matched sibling donor. Haploidentical transplants were performed in 40 patients (22.3%) and MUD transplants in 33 patients (18.4%) respectively. In this study 145 patients (81%) were treated with MAC regimens and the remaining 34 patients (19%) underwent transplantation with RIC and NMA regimens. Both groups were homogeneous according to transplant type (*p* = 0.53). MAC was performed to 84% of the treosulfan arm while 77.6% to the busulfan arm (*p* = 0.36). When we analyzed the pre‐transplant risk factors, the median HCT CI score was 1 in the busulfan group while it was 0 in the treosulfan group (*p* < 0.001). The median EBMT risk scores were 3 and 4 for busulfan and treosulfan respectively (*p* = 0.03). The distribution of malignancy types is shown in Table 2.

The pre‐transplant remission status was not homogeneously distributed among both groups. The percentage of patients who were transplanted in first complete remission (CR1) were higher in the treosulfan (59.6%) group compared to busulfan (34.1%). Reciprocally, transplantation in second complete remission (CR2) was higher in the busulfan (44.7%) group compared to the treosulfan group (22.3%). Apart from the distinct distributions of CR1 and CR2 (*p* = 0.006), the distributions of patients transplanted in partial remission, active disease and in third remission were similar. The drugs administered for GVHD prophylaxis are depicted in Table 2. While CsA/MTX was the most commonly used prophylaxis in the busulfan group, TACRO/MMF/PTCy was the most commonly used prophylaxis in the treosulfan group (*p* = 0.024).

### 3.2 Primary outcomes

The median follow up was 14 months for the treosulfan group while it was 11 months for the busulfan group (*p* = 0.16) (Table 3). RRT, which was monitored during the hospital stay and 3 months after discharge were similar between two groups. Among the treosulfan group, RRT was detected in 11.7% of the patients, while the detection rate of RRT was 7.1% within the busulfan group. None of the patients in the treosulfan group experienced seizures and of the two patients who developed CNS complications, one experienced intracranial hemorrhage while the other patient was diagnosed with calcineurin inhibitor associated posterior encephalopathy. Among patients who received busulfan, 3 three patients developed CNS complications. Intracranial hemorrhage was encountered in two patients while one patient was diagnosed with thrombotic microangiopathy. The NRM was 35.5% in the treosulfan group while it was 29.4% in the busulfan group (*p* = 0.962). The most common cause of NRM was sepsis for both groups.

**TABLE 3:** Clinical outcomes of patients who received treosulfan or busulfan based conditioning regimens.

	Treosulfan *n* = 94	Busulfan *n* = 85	*p*‐Value
Median follow up (months)	14	11	0.16
Median survival (months)	30	11	0.04
Regimen related toxicity			
Encountered *n* (%)	11 (11.7)	9 (10.5)	0.4
Cardiac *n* (%)	2 (2.1)	0 (0)	
CNS complication *n* (%)	2 (2.1)	3 (3.5)	
Renal *n* (%)	0 (0)	1 (1.1)	
Hepatic *n* (%)	2 (2.1)	0 (0)	
Pulmonary *n* (%)	2 (2.1)	1 (1.2)	
Hemorrhagic cystitis *n* (%)	1 (1.1)	4 (4.7)	
Combined *n* (%)	2 (2.1)	0 (0)	
*Non‐relapse mortality n* (*%*)	34 (36.1)	25 (29.4)	962
Sepsis *n* (%)	19 (20.2)	14 (16.4)	
GVHD *n* (%)	11 (11.7)	5 (5.8)	
Engraftment failure *n* (%)	1 (1.1)	3 (3.5)	
Non‐infectious CNS complication *n* (%)	0 (0)	3 (3.5)	
MOF *n* (%)	2 (2.1)	0 (0)	
Relapse related mortality *n* (%)	16 (17)	29 (34)	
Extensive Chronic GVHD *n* (%)	13 (15.7)	27 (32.1)	<0.001
Skin *n* (%)	3 (3.6)	19 (22.6)	<0.001
Gastrointestinal *n* (%)	0 (0)	2 (2.4)	
Sicca like syndrome *n* (%)	2 (2.1)	0 (0)	
Hospitalization days (mean)	29.3	28.1	0.15
Neutrophil engraftment, mean days (SD)	16.1 (2.6)	15.3 (4.8)	0.1
Platelet engraftment, mean days	21.1	18.6	0.08
Severe systemic infection during neutropenic fever			
Encountered *n* (%)	15 (16)	64 (75.3)	
Infection (CTCAE 3 or higher) *n* (%)	4 (4.3)	57 (67.1)	
ICU requirement *n* (%)	1 (1.1)	2 (2.4)	
ICU requirement and mortality *n* (%)	10 (10.6)	5 (5.8)	
CMV reactivation			
Encountered *n* (%)	74 (78)	34 (60)	0.01
Preemptive therapy *n* (%)	73 (77.7)	44 (51.8)	
Treatment of syndrome *n* (%)	1 (1.1)	7 (8.2)	
SOS *n* (%)	2 (2.1)	3 (3.5)	0.66
Fungal Infection			
Not encountered *n* (%)	72 (76.6)	54 (63.5)	
Probable/Possible *n* (%)	9 (9.6)	21 (24.7)	
Proven *n* (%)	13 (13.8)	10 (11.8)	
Acute GVHD (Grade 3 or 4)			
Not encountered *n* (%)	59 (67.8)	59 (69.4)	
Skin *n* (%)	10 (11.5)	10 (11.8)	
Gastrointestinal *n* (%)	11 (12.6)	4 (4.7)	
Skin and gastrointestinal *n* (%)	2 (2.3)	5 (5.9)	
Liver *n* (%)	0 (0)	2 (2.4)	
Multisystemic *n* (%)	4 (4.6)	4 (4.7)	
Sicca like syndrome *n* (%)	1 (1.1)	0 (0)	
BOOP *n* (%)	0 (0)	1 (1.2)	

Abbreviations: BOOP, bronchiolitis obliterans organizing pneumonia; CNS, central nervous system; CTCAE, common terminology criteria for adverse events; GVHD, graft‐versus‐host‐disease; ICU, intensive care unit; MOF, multi‐organ failure; SD, standard deviation; SOS, sinusoidal obstruction syndrome.

### 3.3 Secondary outcomes

**FIGURE 1:**
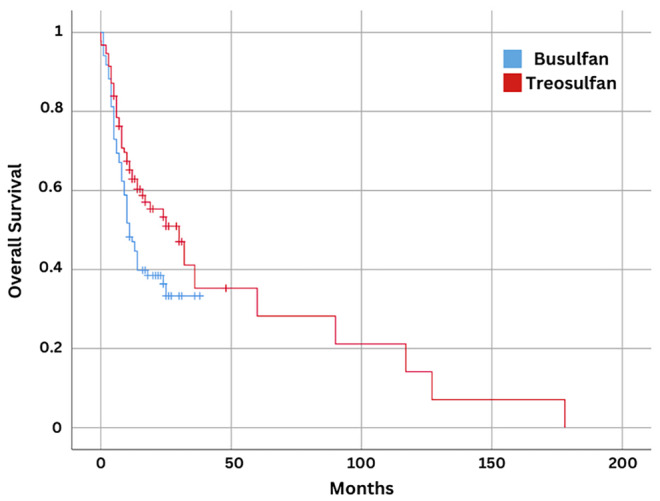
Overall Survival Analysis of Patients Who Underwent Conditioning Regimens With Treosulfan or Busulfan.

Median survival was calculated as 30 months and 11 months for treosulfan and busulfan respectively (*p* = 0.04) (Figure 1). The RRM was 17% in the treosulfan group while it was 34% in the busulfan group. The incidence of extensive chronic GVHD was 15.7% in the treosulfan group compared to 32.1% in the busulfan group and there was a statistically significant difference between two groups (*p* < 0.001). Similarly, chronic skin GVHD was observed in 3.6% of the patients who received treosulfan while it was seen in 22.6% of the busulfan group (*p* < 0.001). The remaining subgroups of chronic GVHD types were not statistically different among two groups.

Mean values of the total days of hospitalization were 29.3 and 28.1 for treosulfan group and busulfan group respectively (*p* = 0.15). Mean duration of neutropenia was longer in the treosulfan group (16.1) compared to the busulfan group (15.3) (*p* = 0.1). Similarly, platelet engraftment was achieved later in patients who received treosulfan (mean days 21.1) compared to busulfan (mean days 18.6), however; a notable clinical importance was not observed (*p* = 0.15). Neutropenic fever was encountered in all patients however, more serious infections (CTCAE grade 3 or higher) were more frequent in the busulfan group (75.4%) compared to the treosulfan group (16%). Among the 15 patients who were diagnosed with serious infection within the treosulfan group, 10 patients died in the ICU due to infection. Within the busulfan group, among the 64 patients who were diagnosed with serious infection within the treosulfan group, 5 patients died in the ICU due to infection.

Donor chimerism at Day 30 and Day 100 were similar within two groups. Donor chimerism was achieved in all patients on Day 30 in both groups (mean chimerism was 99.3% vs. 99.2% for treosulfan and busulfan respectively). Donor chimerism at Day 100 was not achieved in one patient in each group. Detection of CMV reactivation within the follow up period was higher in the treosulfan group (79.7%) compared to busulfan group (60%), therefore a higher percentage within the treosulfan group received preemptive treatment due to serological activation. However, patients who required CMV disease treatment who manifested symptoms like fever, pneumonia, hepatitis, gastroenteritis or encephalitis were higher in the busulfan group (8.3%) as opposed to the treosulfan group (1.1%).

SOS was documented in two patients in the treosulfan group while it was documented in three patients who received busulfan. Fungal infection was observed in 23.4% and 36.5% of the patients in treosulfan and busulfan groups respectively. The incidence of acute GVHD (Grade 3 or higher) was 32.2% and 31.6% in treosulfan and busulfan groups respectively.

## 4 DISCUSSION

We present the outcomes of treosulfan based conditioning regimen and busulfan based regimen in adult patients who underwent allogeneic HSCT and showed that RRT and NRM were similar between the two groups. Results of our real life data showed that treosulfan is an effective and reliable choice in HSCT with noninferior outcomes compared to busulfan. Since there is limited data on the outcomes of treosulfan based regimens in adult patient population from our country, we chose to investigate the performance of the regimen in a transplant center in which nonmyeloablative, myeloablative, MUD and haploidentical HSCT was executed. Our main objective was to demonstrate the outcomes of the treosulfan based regimen however, as we had a similar patient population who were administered busulfan in the past decade with high SOS risk before the treosulfan regime, we chose to do a historical comparison.

Even though the distribution of diagnoses is homogeneous in both groups, there are important differences between them especially in terms of GVHD prophylaxis. The following outcome comparison analysis therefore should not be regarded as a prospective comparison study and we will mention the discordances and discrepancies in the discussion below. Our aim is to convey data regarding regimen‐related toxicity and complications that we encountered related to transplantation while using treosulfan in a standard transplant clinic. Herein, we interpret the results of our current treosulfan experience with our previous busulfan experience and as this is the design of the study, our comparative results cannot depict superiority between two regimens.

Even though there was a difference in distribution according to the malignancy types (MDS, NHL, HL and CML) between the treosulfan and busulfan groups, this was not a clinically notable distinction. This distribution was expected since standard non myeloablative conditioning with treosulfan is preferred in patients with MDS, NHL and HL while busulfan based regimens are preferred in patients with CMPD.

Ruutu and Kröger published two Phase II trials, investigating the effects of treosulfan based reduced intensity regimens used in MDS and ALL patients.^19^, ^20^ The results of these studies were promising and revealed acceptable toxicity and efficacy among the patients who were enrolled in the studies. Later, Nagler and his colleagues reported the long‐term outcome results after treosulfan based conditioning regimens in AML patients who underwent allogeneic transplantation.^13^ In this study, treosulfan was used not only in RIC, but also in MAC regimens and the results suggested that it can be used as an alternative to busulfan in patients treated with myeloablative conditioning due to the incidence of favorable non relapse mortality and low risk of SOS.^21^ Similarly, to these studies, we detected similar RRT incidences and concluded that treosulfan was well tolerated in terms of toxicity. There was no statistically significant difference in NRM between treosulfan and busulfan groups, which sheds light on the safety profile of the drug. Similar findings were reported in a recent meta‐analysis in which treosulfan was found to be noninferior to busulfan in terms of NRM.^22^

Chronic GVHD was encountered more frequently in the busulfan group (32.1% vs. 15.7%) and this difference was most prominently observed in the skin GVHD subtype. The post‐inflammatory state which occurs after the damage caused by busulfan within the early post‐transplant period may set a scene for the establishment of chronic skin GVHD.^23^, ^24^, ^25^ Chronic GVHD prevalence was found to be similar in a recent meta‐analysis that compared treosulfan and busulfan based regimens in patients with AML/MDS.^21^ As usage of treosulfan became more common with the research in recent years, we implemented a strategy that prioritized treosulfan usage in our clinic. Since post‐transplant cyclophosphamide for GVHD prophylaxis is also a relatively newer practice, we believe that the combination of treosulfan with post‐transplant cyclophosphamide may contribute to the lower incidence of chronic GVHD. The difference in the regimens used for GVHD prophylaxis, which may have impacted our results is also another limitation of our study. Treosulfan was found to be non‐inferior to busulfan in terms of days of hospitalization, donor chimerism, thrombocyte engraftment and neutrophil engraftment periods.

Treosulfan was found to be superior to busulfan in terms of EFS and OS in older AML or MDS patients in a recent Phase 3 trial.^26^ As both groups included patients with various hematological malignancies, we did not include overall survival as an outcome in our study. However, 30 months of median survival was estimated for the treosulfan group compared to 11 months for the busulfan group (*p* = 0.04). We believe that this statistical significance might be due to the low number of patients in our study and this outcome should not be regarded as a data to implement in real life. Due to the limited number of patients, we did not compare subgroups of specific diagnoses. We did not encounter a significant difference after investigating the median survival graphs of each cohort within both groups.

As busulfan crosses the blood brain barrier and decreases the seizure threshold, patients receiving busulfan are also given prophylactic antiepileptics like benzodiazepines, levetiracetam or phenytoin.^27^ Addition of anti‐epileptics may cause side effects along with the increased risk of drug interactions. In coherence with current literature, without necessitating any prophylactic antiepileptics, we did not encounter serious CNS complications like seizures in the treosulfan group. Our study was concordant with Naglers study, in which long term effects of treosulfan was investigated, where treosulfan was found to be safe in term of CNS toxicity.^13^ As seizure prophylaxis was administered to all patients in the busulfan group, epilepsy was not observed in any of the patients in the busulfan group. We believe that the reason for the CNS complications of the 3 patients who were administered busulfan may have been attributed to thrombocytopenia or transplant immunology.

The incidence of serious infections (CTCAE 3 or higher) was significantly higher in the busulfan group, however; the percentage of patients who died due to infection was higher in the treosulfan group. This may be due to the sustained immune ablative features of treosulfan. As the busulfan group included more of the TR2 or higher group, the heavily pretreated patient population may be a confounding factor which could explain the higher incidence of infections.

The median HCT‐CI score of the patients in the treosulfan group was 0 while it was 1 in the busulfan group. Even though the difference between HCT‐CI scores were statistically significant, since both 0 and 1 are low scores, we believe the effect of the comorbidity burden was negligible. The median EBMT risk scores of the treosulfan and busulfan groups were 4 and 3 respectively and similarly we cannot make a deduction on how this difference may have affected the results of our study.

Since it was shown in mice that treosulfan was more effective in T and B cell ablation compared to busulfan, we also aimed to investigate the incidence of CMV reactivation and infections.^5^ The patients who were given preemptive CMV treatment were higher in the treosulfan group but the patients who manifested systemic symptoms of CMV and were treated for systemic CMV infection was higher in the busulfan group. Patients within the busulfan groups may have been detected at a later disease stage, as the intervals between CMV follow up tests were longer between 2016 and 2019, and most of the busulfan patients were transplanted within this period. We believe this may explain the lower percentage of patients within the busulfan group who received preemptive treatment. This is our observation and is one of the limitations of our study which could be further investigated in future trials.

One of the differences which has clinical implications is that busulfan is administered four times a day while treosulfan is administered as a single dose daily.^28^ A steady‐state plasma concentration of busulfan is difficult to achieve due to high variability in genetic polymorphisms and patient features like fat mass.^29^ Exposure to higher busulfan concentrations can increase the risk of GVHD, transplant related infections and SOS.^30^ Many centers that use busulfan based conditioning regimens use prophylactic low molecular heparin or defibrotide in patients at high risk for SOS which creates additional pharmacological, clinical and financial burden.^6^ The incidence of SOS was similar within two groups however treosulfan was more advantageous in terms of clinical practice. Regardless of their SOS risk, prophylactic defibrotide was not administered to any patient in the treosulfan group. As all the patients in the busulfan group had high risk for SOS, they received prophylactic defibrotide. SOS was encountered in only two patients in the treosulfan group, despite the lack of prophylactic defibrotide and our results are parallel to the current studies that underline the safety of treosulfan in terms of SOS.^31^ Treosulfan is administered at a fixed dose according to the body surface area and the incidence of SOS is low as previously demonstrated in many trials, thus the above mentioned prophylaxis done for busulfan is not necessary. Adding to the cost benefit of not administering prophylactic defibrotide, eliminating a drug from the regimen decreases the drug interaction risk for patients who are already at high risk due to multidrug treatment.

Adding on to the lack of previous research on this topic, this study has several limitations. Firstly, this is a retrospective analysis with a relatively small sample size for the comparison of drug efficacies. Nevertheless, due to the scarcity of single center real life data like our study, we believe that data derived with the analysis of this patient group is valuable and this study should pave the way for more research to come.

When performing HSCT with MAC or RIC, treosulfan can be safely administered to patients with MUD or haploidentical transplantation and randomized controlled research on its effectiveness and safety profile is imminently needed. To conclude, our results indicate that treosulfan based regimen is as safe and effective as busulfan based regimen in allogeneic HSCT in terms of RRM, NRM and GVHD therefore can be considered as a safe and efficacious alternative.

## ETHICAL APPROVAL

The study was approved by the Institutional Review Board of Acıbadem Mehmet Ali Aydınlar University School of Medicine (ATADEK) with decree number 2023–03/84.

## AUTHOR CONTRIBUTIONS

**Ant Uzay:** Conceptualization (lead); investigation (equal); methodology (lead); project administration (supporting); resources (supporting); supervision (supporting); writing – original draft (supporting); writing – review and editing (equal). **Yasemin Gündoğdu:** Data curation (equal); formal analysis (equal); resources (equal); writing – original draft (lead); writing – review and editing (equal). **Barış Koşan:** Data curation (equal); methodology (supporting); project administration (equal); resources (equal); software (equal); writing – original draft (equal); writing – review and editing (supporting). **Tuğba Yetiş:** Data curation (equal); project administration (supporting); software (lead); visualization (equal); writing – original draft (equal). **S. Sami Kartı:** Conceptualization (equal); methodology (supporting); project administration (equal); supervision (lead); writing – review and editing (equal).

## FUNDING INFORMATION

This research did not receive any grant from funding agencies in the public, commercial, or not‐for‐profit sectors.

## CONFLICT OF INTEREST STATEMENT

The authors declare that they have no known competing interests or personal relationships that could have influenced the work reported in this study.

## Data Availability

The database analyzed during the current study is available from the corresponding author on reasonable request.

## References

[1] Styczyński J , Tridello G , Koster L , et al. Death after hematopoietic stem cell transplantation: changes over calendar year time, infections and associated factors. Bone Marrow Transplant. 2020;55(1):126‐136. doi:10.1038/s41409-019-0624-z 31455899 PMC6957465

[2] Malagola M , Polverelli N , Rubini V , et al. GITMO registry study on allogeneic transplantation in patients aged ≥60 years from 2000 to 2017: improvements and criticisms'. Transplant Cell Ther. 2022;28(2):2. doi:10.1016/j.jtct.2021.11.006 96.e1, 96.e11. 34818581

[3] Gyurkocza B , Sandmaier BM . Conditioning regimens for hematopoietic cell transplantation: one size does not fit all. Blood. 2014;124(3):344‐353. doi:10.1182/blood-2014-02-514778 24914142 PMC4102707

[4] Casper J , Knauf W , Kiefer T , et al. Treosulfan and fludarabine: a new toxicity‐reduced conditioning regimen for allogeneic hematopoietic stem cell transplantation. Blood. 2004;103(2):725‐731. doi:10.1182/blood-2002-11-3615 12947008

[5] Sjöö F , Hassan Z , Abedi‐Valugerdi M , et al. Myeloablative and immunosuppressive properties of treosulfan in mice. Exp Hematol. 2006;34(1):115‐121. doi:10.1016/j.exphem.2005.09.015 16413398

[6] Carreras E et al. The EBMT Handbook Hematopoietic Stem Cell Transplantation. Springer International Publishing; 2018.

[7] Center for Drug Evaluation and Research . (no date) *Defitelio* (*defibrotide sodium*), *U.S. Food and Drug Administration* . https://www.fda.gov/drugs/resources‐information‐approved‐drugs/defitelio‐defibrotide‐sodium

[8] Chaguaceda C , Aguilera‐Jiménez V , Gutierrez G , Roura J , Riu G . Oral levetiracetam for prevention of busulfan‐induced seizures in adult hematopoietic cell transplant. Int J Clin Pharm. 2020;42(2):351‐354. doi:10.1007/s11096-020-00977-7 32026356

[9] Bacigalupo A , Ballen K , Rizzo D , et al. Defining the intensity of conditioning regimens: working definitions. Biol Blood Marrow Transplant. 2009;15(12):1628‐1633. doi:10.1016/j.bbmt.2009.07.004 19896087 PMC2861656

[10] Shimoni A , Labopin M , Savani B , et al. Intravenous busulfan compared with treosulfan‐based conditioning for allogeneic stem cell transplantation in acute myeloid leukemia: a study on behalf of the acute leukemia working Party of European Society for blood and marrow transplantation. Biol Blood Marrow Transplant. 2018;24(4):751‐757. doi:10.1016/j.bbmt.2017.12.776 29247780

[11] Bearman SI , Appelbaum FR , Buckner CD , et al. Regimen‐related toxicity in patients undergoing bone marrow transplantation. J Clin Oncol. 1988;6(10):1562‐1568. doi:10.1200/jco.1988.6.10.1562 3049951

[12] Danylesko I , Shimoni A , Nagler A . Treosulfan‐based conditioning before hematopoietic SCT: more than a bu look‐alike. Bone Marrow Transplant. 2011;47(1):5‐14. doi:10.1038/bmt.2011.88 21478921

[13] Nagler A , Labopin M , Beelen D , et al. Long‐term outcome after a treosulfan‐based conditioning regimen for patients with acute myeloid leukemia: a report from the acute leukemia working party of the european society for blood and marrow transplantation. Cancer. 2017;123(14):2671‐2679. doi:10.1002/cncr.30646 28329410

[14] Glucksberg H , Storb R , Fefer A , et al. Clinical manifestations of graft‐versus‐host disease in human recipients of marrow from HL‐a‐matched sibling donors. Transplantation. 1974;18(4):295‐304. doi:10.1097/00007890-197410000-00001 4153799

[15] MacMillan ML , DeFor TE , Weisdorf DJ . What predicts high risk acute graft‐versus‐host disease (GVHD) at onset?: identification of those at highest risk by a novel acute GVHD risk score. Br J Haematol. 2012;157(6):732‐741. doi:10.1111/j.1365-2141.2012.09114.x 22486355 PMC3654854

[16] Jagasia MH , Greinix HT , Arora M , et al. ‘National Institutes of Health consensus development project on criteria for clinical trials in chronic graft‐versus‐host disease: I. The 2014 diagnosis and staging working group report’. Biol Blood Marrow Transplant. 2015;21(3):389‐401.e1. doi:10.1016/j.bbmt.2014.12.001 25529383 PMC4329079

[17] *Common terminology criteria for adverse events* (*CTCAE*). 2017 https://ctep.cancer.gov/protocoldevelopment/electronic_applications/docs/ctcae_v5_quick_reference_5x7.pdf 15818867

[18] Donnelly JP , Chen SC , Kauffman CA , et al. Revision and update of the consensus definitions of invasive fungal disease from the European organization for research and treatment of cancer and the mycoses study group education and research consortium. Clin Infect Dis. 2019;71(6):1367‐1376. doi:10.1093/cid/ciz1008 PMC7486838 31802125

[19] Ruutu T , Volin L , Beelen DW , et al. Reduced‐toxicity conditioning with treosulfan and fludarabine in allogeneic hematopoietic stem cell transplantation for myelodysplastic syndromes: final results of an international prospective phase II trial. Haematologica. 2011;96(9):1344‐1350. doi:10.3324/haematol.2011.043810 21659356 PMC3166105

[20] Kröger N , Bornhäuser M , Stelljes M , et al. Allogeneic stem cell transplantation after conditioning with treosulfan, etoposide and cyclophosphamide for patients with all: a phase II‐study on behalf of the German cooperative transplant study group and all study group (GMALL). Bone Marrow Transplant. 2015;50(12):1503‐1507. doi:10.1038/bmt.2015.202 26367236

[21] Zhu S , Liu G , Liu J , Chen Q , Wang Z . Long‐term outcomes of treosulfan‐ vs. busulfan‐based conditioning regimen for patients with myelodysplastic syndrome and acute myeloid leukemia before hematopoietic cell transplantation: a systematic review and meta‐analysis. Front Oncol. 2020;10:2, 4‐6. doi:10.3389/fonc.2020.591363 33425740 PMC7793760

[22] Strong Rodrigues K , Oliveira‐Ribeiro C , de Abreu Fiuza Gomes S , Knobler R . Cutaneous graft‐versus‐host disease: diagnosis and treatment. Am J Clin Dermatol. 2017;19(1):33‐50. doi:10.1007/s40257-017-0306-9 PMC5797560 28656563

[23] Sennett R , Jama BM , Hinds B , Tzachanis D , Morris GP , Marsch AF . Local immune cell infiltration in cutaneous acute graft versus host disease. Int J Womens Dermatol. 2020;6(4):311‐317. doi:10.1016/j.ijwd.2020.05.009 33015293 PMC7522857

[24] Hill GR . Inflammation and bone marrow transplantation. Biol Blood Marrow Transplant. 2009;15(1):139‐141. doi:10.1016/j.bbmt.2008.11.008 19147094

[25] Beelen DW , Stelljes M , Reményi P , et al. Treosulfan compared with reduced‐intensity busulfan improves allogeneic hematopoietic cell transplantation outcomes of older acute myeloid leukemia and myelodysplastic syndrome patients: final analysis of a prospective randomized trial. Am J Hematol. 2022;97(8):1023‐1034. doi:10.1002/ajh.26620 35617104

[26] McCune JS , Wang T , Bo‐Subait K , et al. Association of antiepileptic medications with outcomes after allogeneic hematopoietic cell transplantation with busulfan/cyclophosphamide conditioning. Biol Blood Marrow Transplant. 2019;25(7):1424‐1431. doi:10.1016/j.bbmt.2019.03.001 30871976 PMC6615968

[27] Seydoux C , Battegay R , Halter J , et al. Impact of busulfan pharmacokinetics on outcome in adult patients receiving an allogeneic hematopoietic cell transplantation. Bone Marrow Transplant. 2022;57(6):903‐910. doi:10.1038/s41409-022-01641-6 35361896 PMC9200635

[28] Du X , Huang C , Xue L , et al. The correlation between busulfan exposure and clinical outcomes in Chinese pediatric patients: a population pharmacokinetic study. Front Pharmacol. 2022;13:2. doi:10.3389/fphar.2022.905879 PMC9243314 35784763

[29] Andersson BS , Thall PF , Madden T , et al. Busulfan systemic exposure relative to regimen‐related toxicity and acute graft‐versus‐host disease: defining a therapeutic window for I.V. bucy2 in chronic myelogenous leukemia. Biol Blood Marrow Transplant. 2002;8(9):477‐485. doi:10.1053/bbmt.2002.v8.pm12374452 12374452

[30] Greystoke B , Bonanomi S , Carr TF , et al. Treosulfan‐containing regimens achieve high rates of engraftment associated with low transplant morbidity and mortality in children with non‐malignant disease and significant co‐morbidities. Br J Haematol. 2008;142(2):257‐262. doi:10.1111/j.1365-2141.2008.07064.x 18492115

[31] Slatter MA , Rao K , Amrolia P , et al. Treosulfan‐based conditioning regimens for hematopoietic stem cell transplantation in children with primary immunodeficiency: United Kingdom experience. Blood. 2011;117(16):4367‐4375. doi:10.1182/blood-2010-10-312082 21325599

